# Cytokine responses and correlations thereof with clinical profiles in children with enterovirus 71 infections

**DOI:** 10.1186/s12879-015-0965-1

**Published:** 2015-06-11

**Authors:** Ning Ye, Xun Gong, Li-li Pang, Wen-juan Gao, Ya-ting Zhang, Xiao-le Li, Na Liu, Dan-di Li, Yu Jin, Zhao-jun Duan

**Affiliations:** Medical School of Nanjing University, Nanjing Children’s Hospital, Nanjing, 210093 People’s Republic of China; National Institute for Viral Disease Control and Prevention, China CDC, Chang-Bai Rd 155, Beijing, 100052 People’s Republic of China; Guang’anmen Hospital, China Academy of Chinese Medical Sciences, Beijing, 100053 People’s Republic of China

**Keywords:** Enterovirus 71, Infection, Cytokines, Inflammation

## Abstract

**Background:**

Severe complications associated with EV71 infections caused many infants death. However, the pathogenesis of EV71 infection in the severe cases remained poorly understood.

**Methods:**

In this study we collected plasma and cerebrospinal fluid (CSF) specimens drawn in the acute and/or recovery phases from EV71-infected individuals, and plasma specimens from healthy children served as normal controls. We compared the levels of cytokines and chemokines determined by a Luminex-based cytokine bead array.

**Results:**

The plasma levels of IL-1β and IL-6 were significantly higher in severe and critical cases than in mild patients and normal controls. Higher plasma levels of IL-6, IL-10, and IL-8 were evident in critical than severe cases. The CSF levels of IL-6, IL-8, and IP-10 were higher, and that of RANTES lower (compared to plasma), in severe and critical patients. Significantly lower CSF levels of cytokines and chemokines were recorded in the recovery than the acute phase in severe and critical cases treated with intravenous immunoglobulin (IVIG) and glucocorticoids. Only the CSF levels of IL-6, IP-10, and IL-8 were significantly correlated with white blood cell counts, and absolute neutrophil and monocyte counts, in severe cases. Furthermore, the CSF levels of IL-6 were correlated with temperature in both cases.

**Conclusions:**

These data indicate that a major cytokine response and inflammation, in both plasma and the CNS, are features of disease caused by EV71 infection. Systemic inflammation caused by EV71 infection exacerbated the deterioration of the disease, and resulted in the disease progression to the critical illness stage.

## Background

Outbreaks of enterovirus 71 (EV71) have been reported in Bulgaria (1975), Hungary (1978), Malaysia (1997), Taiwan (1998), China (2008), and Korea (2009), in the time since the virus was first isolated from patients with central nervous system (CNS) disease in California in 1969 [1-5]. In China, the first nationwide epidemic of hand, foot, and mouth disease (HFMD) was associated with an EV71 outbreak in 2008 in Anhui province. HFMD epidemics continue to occur to the present day, with apparent increases in severity [6]. During 2013, the Chinese National Health and Family Planning Commission (NHFPC) reported a total of 1,855,559 HFMD cases, including 260 that were fatal.

EV71 is a single-stranded positive-sense RNA virus of the *Picornaviridae* family. Uncomplicated hand, foot, and mouth disease, or herpangina, is the principal clinical manifestation in most patients with EV71 infection. Severe CNS disease and complications including encephalitis, aseptic meningitis, and brain stem encephalitis, are associated with EV71 infections in severely ill patients [1]. EV71-infected patients may succumb to respiratory failure caused by pulmonary edema (PE), followed by circulatory collapse, after CNS injury [7]. Although the pathogenesis of EV71 infection is not well-defined, direct viral-mediated neuropathic damage and indirect immune-mediated effects are considered to have an impact [8]. Previous studies have shown that the severity of clinical manifestations associated with EV71 infection possibly depends on the host immune inflammatory response, including acute cytokine and chemokine storms in the blood and cerebrospinal fluid (CSF) [9-13]. Immune disorder caused by EV71 infection such as elevated proinflammatory cytokine and chemokine may play an important role in the disease outcome of HFMD. Several cytokines and chemokines, including tumor necrosis factor α (TNF-α), IL-1β,-6, −10,-8 and-13 and IFN-γ, were indicated to be associated with brainstem encephalitis (BE) and pulmonary edema (PE) caused by EV71 infection in the previous studies [9,14,15].

Specific therapies for targeting EV71 are under development. On the basis that hyperinflammation plays a role in EV71 pathogenesis, intravenous immunoglobulin (IVIG) and glucocorticoids have been recommended to treat severe EV71 CNS infections. IVIG is a polyclonal preparation from human serum and has been used to treat many viral infections. Previous work has demonstrated that, after IVIG administration, the plasma levels of cytokines including IL-8 and IL-10 decreased significantly in patients with PE [16]. Glucocorticoids are used extensively to treat severe infectious diseases in China, but their efficacy remains controversial. One study found that the levels of many serum cytokines in HFMD patients treated with methylprednisolone did not differ significantly from those of untreated patients [17]. However, no paired comparison of CSF cytokine profiles between patients in the acute and recovery phases, after administration of IVIG and glucocorticoid, has yet been performed.

In the present study, we explored the diversity of cytokines in plasma and CNS specimens from different groups of patients diagnosed with HFMD. Changes in cytokine and chemokine levels were measured in EV71-infected patients given IVIG and glucocorticoid. Moreover, we also determined the correlations between cytokine levels and markers of inflammation including temperature, white blood cell (WBC) counts, or individual counts of neutrophils, lymphocytes, or monocytes.

## Methods

### Patient enrollment

This study was approved by the Ethics Committee of Nanjing Children’s Hospital, and informed written consent was obtained from all legal guardians. Plasma and CSF specimens from individuals with HFMD were collected from April 2010 to May 2012 from Nanjing Children’s Hospital. All (n = 93) patients were confirmed to have EV71 infections using EV71-specific RT-PCR assay of throat swab specimens and/or evidence of EV71-specific IgM-positivity at the time of disease onset. Patients with Coxsackievirus A16 (CA16) infection will be excluded *via* RT-PCR. The primer sequences were: EV71 (sense) 5′-GCAGCCCAAAAGAACTTCAC-3′ and EV71 (antisense) 5′-ATTTCAGCAGCTTGGAGTGC-3′; and CA16 (sense) 5′-ATTGGTGCTCCCACTACAGC-3′ and CA16 (antisense) 5′-TCAGTGTTGGCAGCTGTAGG-3′. Hospitalized children with EV71 infections received IVIG after admission at a dose of 0.5 g/kg body weight on each of 4 consecutive days. Glucocorticoid was also given after admission, at 1-2-mg meprednisone/kg/day, for at least 4 successive days.

### Study groups

Healthy and EV71-infected children were included in the study. EV71-infected patients were divided into a mildly ill group, a severely ill group, and a critically ill group, using criteria of the Handbook for Treatment of HFMD (2010) prepared by the NHFPC [18]. Mild cases were diagnosed with HFMD, with or without fever. Severe cases exhibited obvious symptoms of CNS involvement, as confirmed by the presence of clinical features including lethargy, irritability, headache, decreased reflex and muscle strength, myoclonus, ataxia, nystagmus, oculomotor palsy, and acute limb weakness, with or without neuroimaging. Critical cases were diagnosed when any one of the following symptoms was present: 1) coma with cerebral hernia, 2) respiratory failure or, 3) circulatory collapse. All severe cases and critical cases, who were diagnosed by obvious symptoms of CNS involvement, were treated with IVIG and/or glucocorticoid according to the Handbook for Treatment of HFMD.

### Specimen collection

Plasma and CSF specimens from EV71-infected patients in the acute phase of disease were collected within 6 h of admission, and clinical manifestations commenced within 3 days. Recovery-phase specimens were collected after 7–14 days of treatment with IVIG and glucocorticoid. A total of 104 acute-and recovery-phase CSF specimens were obtained from 52 severely ill patients subjected to repeated lumbar puncture. Nine acute-and five recovery-phase CSF samples were obtained from 9 critical cases (two children died), and all were negative upon bacterial culture. Plasma specimens were obtained from hospitalized children with EV71 infections, including 16 mildly ill, 25 severely ill, and 12 critically ill cases (three of the latter children died). Not all such specimens came from the same patients because of difficulties associated with specimen collection, 14 severe cases and 7 critical cases provided both plasma and CSF specimens. Fifteen plasma specimens from healthy children served as normal controls. These children attended the Nanjing Children’s Hospital Pediatric Primary Care Center for routine examinations, and were confirmed to be free of EV71 or CA16 infections *via* RT-PCR. All plasma and CSF specimens were stored at-80 °C prior to analysis.

### Cytokine assays

The levels of the proinflammatory cytokines IL-1β, IL-6, and tumor necrosis factor α (TNF-α); that of the anti-inflammatory cytokine IL-10; and those of the chemokines interferon gamma-induced protein 10 (IP-10), IL-8, regulated on activation, normal T cell expressed and secreted chemokines (RANTES), monocyte chemotactic protein 1 (MCP-1), and monokine induced by IFN-γ (MIG), in both plasma and CSF, were determined using a cytometric bead array (BD Biosciences, San Diego, CA, USA) employing flow cytometry in accordance with the manufacturer’s protocol. The results are presented as means of data derived from duplicate tests. Data were analyzed using the BD Cytometric Bead Array (software version 1.4). Theoretical limits of detection are shown in parentheses: IL-1β (2.3 pg/ml), IL-6 (1.6 pg/ml), TNF-α (1.2 pg/ml), IL-10 (4.7 pg/ml), IP-10 (0.5 pg/ml), IL-8 (1.2 pg/ml), RANTES (0.002 pg/ml), MCP-1 (1.3 pg/ml), and MIG (1.1 pg/ml).

### Statistical analysis

Concentrations of cytokines and chemokines in plasma and CSF specimens were compared after log transformation of data to increase statistical validity. Data are expressed as medians (with ranges). Cytokine and chemokine levels below the limits of detection were considered to be 10^0^ pg/ml for the purpose of statistical analysis. Between-and among-group comparisons of cytokine and chemokine levels were performed using the Mann–Whitney rank-sum test and Kruskal-Wallis test. The significance of differences in CSF levels between acute-and recovery-phase samples was evaluated using the paired-samples *t*-test. Correlations between cytokine and chemokine levels, on the one hand, and immunological cell counts in CSF specimens, on the other, were calculated using Spearman’s rank correlation (to yield Pearson’s correlation coefficients). A difference was considered to be significant when the P value upon two-tailed *t*-testing was less than 0.05. All statistical analyses were performed using the Statistical Package for the Social Sciences (SPSS) version 13.0 software (SPSS Inc., Chicago, IL).

## Results

### Demographic data, clinical characteristics, and plasma cytokine and chemokine levels in HFMD acute disease samples

Demographic data and clinical characteristics, including WBC counts and admission temperature, are shown in Table 1. Plasma levels of the proinflammatory cytokines IL-1β and IL-6, but not those of TNF-α, were significantly higher in the severely and critically ill groups than the mildly ill group and normal controls, as shown by Mann–Whitney rank-sum testing. Plasma levels of IL-6 were significantly higher in critical than severe cases. Higher plasma IL-10 levels were evident in the critically ill group than the other two groups. Plasma levels of chemokine IL-8, but not IP-10, MCP-1, or MIG, were higher in critically ill patients than in all other groups. Moreover, no significant differences in plasma chemokine levels were evident among severely and mildly ill cases and normal controls (Table 1). Although RANTES levels did not vary among groups, very low levels of RANTES (89.7 pg/ml and 312.2 pg/ml) were found in two critically ill patients, suggesting that plasma RANTES expression may be inhibited in some, but not all, critically ill patients.

**Table 1:** Patient characteristics and plasma chemokine and cytokine levels

Characteristic	Normal controls	EV71-infected patients		
		Mildly ill	Severely ill	Critically ill
Number	15	16	25	12
Age (months)	36 (17–54)	34 (12–66)	29 (11–74)	26 (9–42)
Male/female (n)	9:6	9:7	16:9	8:4
Temperature at admission (°C)	–	37.3 (36.8-39.5)	39.1 (38.0-41.0)^b^	39.2 (38.5-41.7) ^b,^
WBC counts in blood (10^9^/L)	–	8.9(3.5-14.1)	10.7(4.3-17.8)^b^	16.5(7.2-41.0) ^b,c^
IL-1β in pla. (log pg/ml)	0 (und.-0.54)	0 (und.-0.48)	0.93 (und.-1.86) ^a,b^	0.78 (und.-1.69) ^a,b^
IL-6 in pla. (log pg/ml)	0 (und.)	0 (und.-0.75)	0.89 (und.-1.62) ^a,b^	1.36(0.67.-1.93) ^a,b,c^
TNF-α in pla. (log pg/ml)	0 (und.)	0 (und.)	0 (und.-0.66)	0 (und.-0.87)
IL-10 in pla. (log pg/ml)	0 (und.)	0 (und.-1.22)	0 (und.-1.61)	1.12 (und.-2.51) ^a,b,c^
IL-8 in pla. (log pg/ml)	0.76 (und.-1.47)	0.87 (und.-1.64)	0.91 (und.-1.77)	1.67 (0.79-2.62) ^a,b,c^
IP-10 in pla. (log pg/ml)	2.25 (1.79-2.83)	2.44 (1.62-3.13)	2.67 (2.21-3.09)	2.59 (2.00-3.17)
MCP-1 in pla. (log pg/ml)	1.47 (1.15-2.33)	1.39 (1.09-2.08)	1.51 (1.28-2.11)	1.78 (1.30-2.73)
MIG in pla. (log pg/ml)	2.39 (1.71-2.62)	2.55 (1.66-2.75)	2.21 (1.31-2.81)	2.67 (1.73-2.95)
RANTES in pla. (log pg/ml)	4.11 (3.07-4.55)	4.17 (3.38-4.60)	4.27 (3.52-4.71)	4.19 (1.95-4.52)

Data are shown as medians (with ranges). Thus, the levels of cytokines and chemokines are shown as median log_10_ pg/ml (again, with ranges). a. The plasma levels of chemokines and cytokines in EV71-infected patients were higher than in healthy controls (P < 0.05). b. Significant differences (P < 0.05) in such concentrations were evident between the mildly ill group and other groups. c. Significant differences (P < 0.05) were also noted between the critically and severely ill groups. Cytokine and chemokine levels below the detection limits were defined to be 10^0^ pg/ml, to allow statistical analysis. Und: undetermined; pla: plasma

### Cytokine and chemokine levels in CSF specimens from EV71-infected patients with CNS complications

**Fig. 1:**
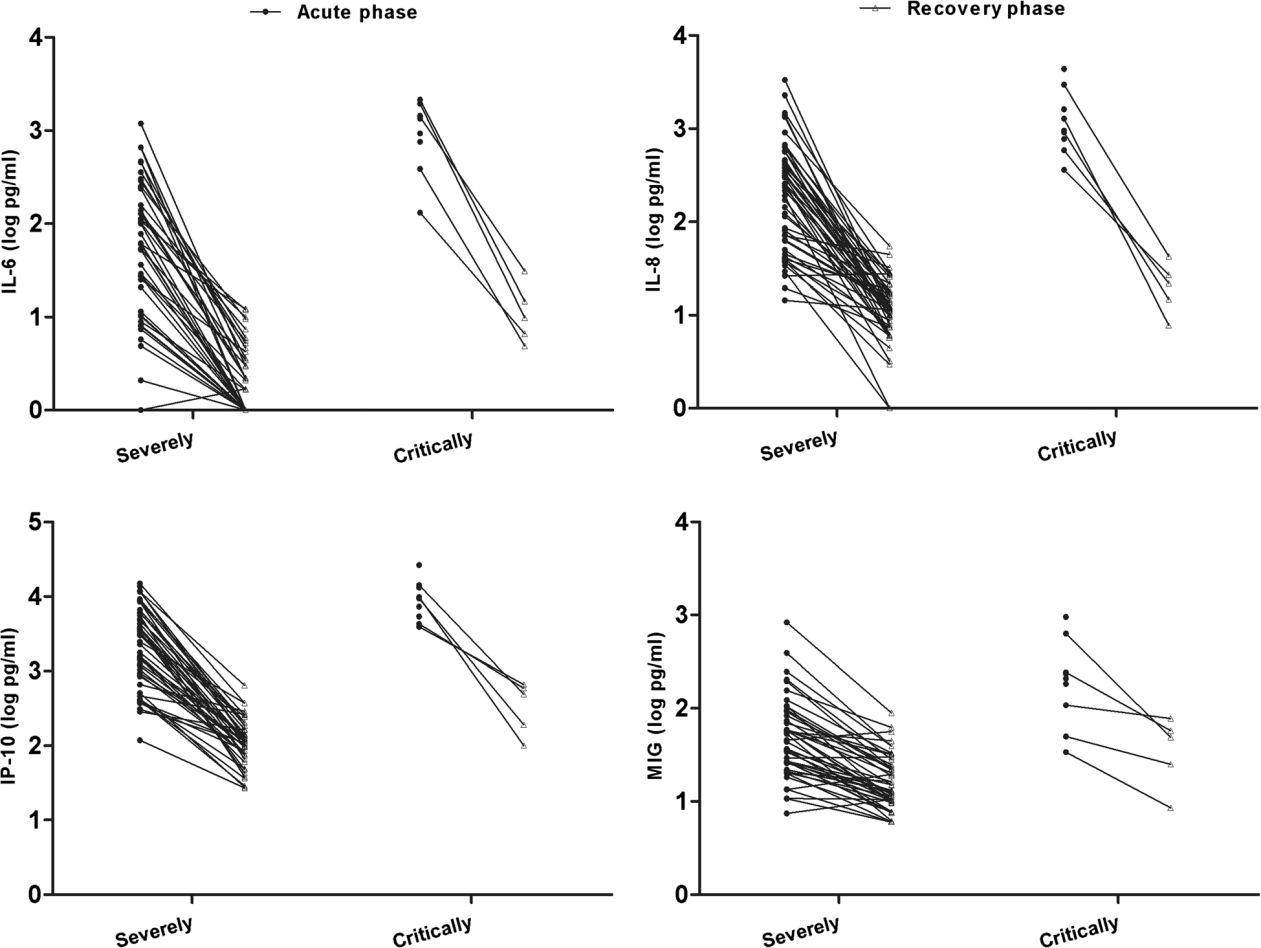
Paired comparisons of data from CSF specimens obtained in the acute and recovery phases. Cytokine and chemokine concentrations in the CSF of EV71-infected patients subjected to repeat lumbar punctures were assayed using the cytometric bead array. Totals of 52 specimens from severely ill patients and 9 from critically ill patients (including 2 deaths) were collected in the acute phase of CNS complications. We also obtained CSF specimens from the 52 severely and 5 critically ill patients in the recovery phase

CSF specimens were obtained from severely and critically ill patients subjected to repeated lumbar puncturing in the acute and recovery phases. Significantly higher levels of IL-6, IP-10, IL-8, and MIG were found in the acute, rather than the recovery, phase upon paired-samples *t*-testing (Fig. 1). The CSF IL-1β levels in the acute phase were close to the detection limit in most severely ill patients, and the CSF IL-1β levels were higher in the acute phase in critically ill patients than observed during the recovery phase. Moreover, no significant change in CSF concentrations of either TNF-α, IL-10, RANTES, or MCP-1 was noted between the acute and recovery phases (data not shown).

Differences between the CSF and plasma levels of cytokines and chemokines in acute-phase samples from patients severely or critically ill with HFMD were analyzed *via t*-testing after log transformation of data. Significant differences in CSF and plasma levels of IL-6, IL-8, IP-10, and RANTES were evident (all P values <0.001). The levels of IL-6, IL-8, and IP-10 were higher in CSF than plasma in severely and critically ill patients. In contrast, significantly lower RANTES CSF levels were evident in severely and critically ill cases (data not shown).

### Correlations between immune cell counts and cytokine levels in CSF collected in the acute phase of illness in patients with CNS complications

**Fig. 2:**
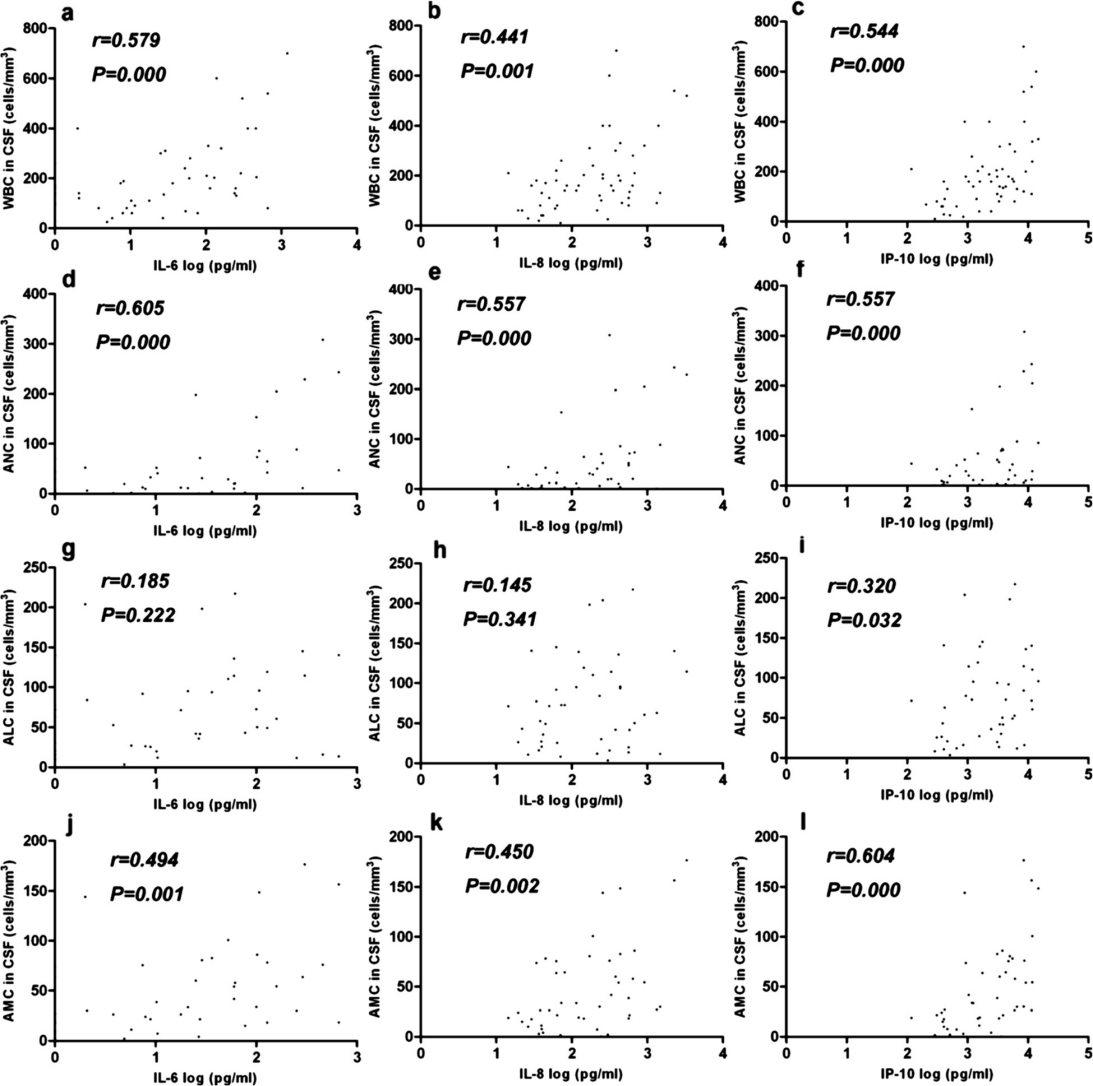
Correlations between CSF cytokine and chemokine levels and immunological cell counts in severely ill patients with EV71 infections. Correlations between IL-6, IL-8, and IP-10 levels; and WBC, ANC, ALC, and AMC counts, were examined in CSF specimens (n = 52). CSF specimens were obtained in the acute phase (on the first day of admission). Spearman’s correlation coefficients (r values) and P values are shown. WBC, white blood cell; ANC, absolute neutrophil count; ALC, absolute lymphocyte count; AMC, absolute monocyte count

**Fig. 3:**
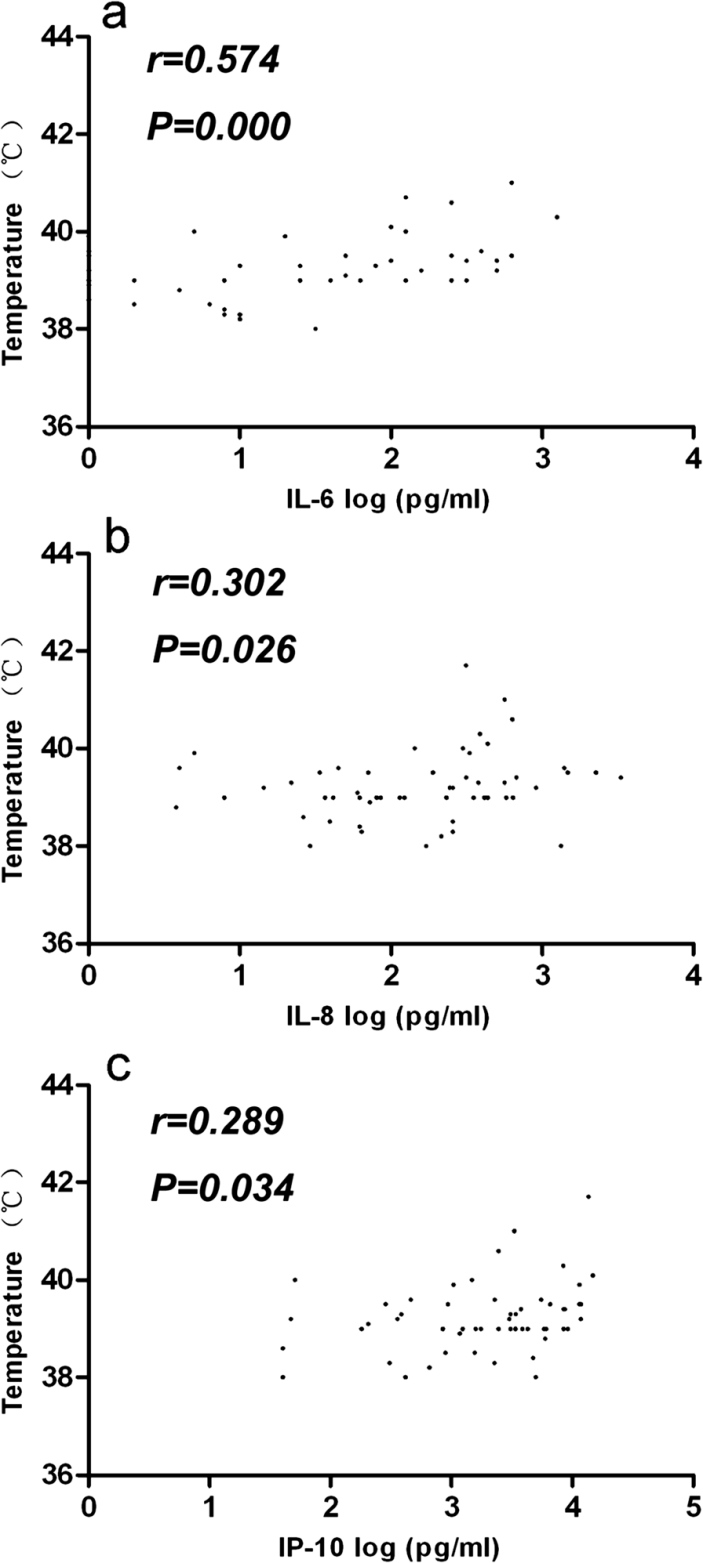
Correlations between CSF cytokine and chemokine levels, and temperature. Correlations between CSF IL-6, IL-8, and IP-10 levels; and temperature, were analyzed. Spearman’s correlation coefficients (r values) and P values: **a** r = 0.574, P = 0.000; **b** r = 0.302, P = 0.026; **c** r = 0.289, P = 0.034

We sought to analyze the correlation between temperature, duration of fever, blood glucose levels, immunological cell counts, and the blood and CSF levels of cytokines and chemokines, in the acute phase of disease in severely and critically ill HFMD patients. As shown in Fig. 2, the CSF levels of IL-6, IL-8 and IP-10 were significantly correlated with CSF WBC counts, absolute neutrophil counts, and absolute monocyte counts, in severe cases. In contrast, no correlation was apparent in critical cases between CSF cytokine levels and the levels of other inflammatory markers. The CSF levels of IL-6 correlated with temperature in EV71-infected patients with CNS complications, as assessed using Spearman’s rank-correlation method (Fig. 3). Correlations between CSF IL-8 and IP-10 levels, and temperature, were also noted, but were not strong (the *r* values were less than 0.5). No correlation was observed between CSF cytokine levels and fever duration; the CSF levels of blood glucose, CRP, or chloride ion; or other biochemical markers. The plasma levels of cytokines and chemokines in the acute phase did not correlate with peripheral blood WBC counts or any other inflammatory markers of EV71-infected patients (data not shown).

## Discussion

Excessive proinflammatory cytokine and chemokine responses were thought to contribute to the severity of EV71 infection [9]. We compared acute-phase CSF concentrations of cytokines and chemokines between severely and critically ill cases. Consistent with previous studies, WBC counts, IL-6 and IL-8 levels in peripheral blood were found to be significantly higher in critically ill than other EV71-infected groups [1,19]. IL-6 and IL-8 are considered to be inducers of inflammatory responses and serve as important markers of inflammation [20]. Elevations in the plasma levels of IL-6 and IL-8 indicated that high-level systemic inflammation developed in critically ill cases, and may play an important role in the pathogenesis of fatal EV71 infection. No differences in the plasma levels of cytokines and chemokines were observed when severely and mildly ill cases were compared. We speculate that disease progresses rapidly when a systemic inflammatory response develops in EV71-infected children. This is consistent with previous study; plasma but not CSF IL-10 levels were significantly higher in critically ill patients but not in others, suggesting that IL-10 was detected at high levels in the blood of only critical cases [10,15]. IL-10 is an important anti-inflammatory cytokine, and development of an intense conflict between an inflammatory and an anti-inflammatory response may play a role in the pathogenesis of critically ill EV71-infected patients. Moreover, although RANTES levels did not differ significantly among groups, it is surprising to observe that the plasma RANTES levels in two critical patients were only ~1 % that of the average of healthy children. High RANTES concentrations effectively inhibit HIV replication [21]. Whether RANTES plays a role in EV71 infection remains unclear; only a limited number of cases have been studied. This question needs to be further investigated.

Enterovirus 71 is considered to be a neurotropic pathogen, injuring the CNS and causing severe clinical outcomes in infected patients [1]. We found that the levels of IL-6, IL-8, and IP-10 were higher in CSF than plasma in both severe and critical cases. Significantly higher CSF levels of cytokines and chemokines, including IL-1β, IL-6, IP-10, IL-8, and MIG, were evident in the acute phases of critical than severe cases. Such results indicate that systemic inflammation progresses in the acute phase of critical illness. On the one hand, such an obvious distinction between the two phases constitutes solid evidence to the effect that elevated CSF cytokine levels play important roles during the acute phase of severe EV71 infection. Significantly lower CSF levels of IL-6, IP-10, IL-8, and MIG were noted during the recovery (compared to the acute) phase of illness in EV71-infected patients. Unlike systemic inflammation, which developed principally in critically ill patients, intensive CNS inflammation (reflected by elevation in cytokine levels and immune cell counts) was universally present in EV71-infected patients, accompanied by CNS disease. In other words, development of a CNS inflammatory response may be essential in induction of CNS complications in EV71-infected patients. More extensive inflammation may adversely affect CNS function [22]. Therefore, proinflammatory cytokines and chemokines may play both beneficial and harmful roles in HFMD associated with EV71 infection. Abnormal CNS inflammatory responses induced by elevated cytokine levels may in part explain the development of neurogenic PE in critically ill EV71-infected patients [14]. Blocking of inflammatory cytokine actions at certain stages of disease may thus be rational (and possible) when EV71 infections are to be managed.

On the other hand, differences in cytokine levels during the recovery compared to the acute phase in severely ill patients may reflect the fact that IVIG and glucocorticoid temper the extensive inflammatory response triggered by EV71 infection. Glucocorticoids are powerful immunosuppressants when given at pharmacological doses, and suppress inflammation by reducing the expression levels of proinflammatory cytokines, while enhancing those of anti-inflammatory cytokines (e.g., IL-10 and TGF-β) [23]. IVIG potentially inhibits the actions of various contributors to inflammation, including proinflammatory cytokines and fragments of complement [24]. Previous studies showed that IVIG effectively treated various forms of viral encephalitis [25-28]. However, the exact roles played by IVIG and glucocorticoids in reducing inflammation caused by EV71 infection remain unclear; it is ethically unfeasible to form control groups that would be required to examine this topic. Further study is needed to determine optimal therapeutic doses and timing of intervention clinically.

Proinflammatory cytokines and chemokines are potent pyrogens, and high fever was more frequent in severely or critically ill patients. We found that IL-6 concentrations in CSF was associated with temperature in severely ill patients and the levels of the IL-6, IL-8 and IP-10, were significantly correlated with WBC counts, absolute neutrophil counts, and absolute monocyte counts in the CSF of severely ill patients. These findings are similar to those of previous studies on enteroviral meningitis, including that caused by echovirus type 4 [29]. Earlier work suggested that, upon EV71 infection, IL-8 and IP-10 were produced principally by CNS microglia or astrocytes, rather than immune cells circulating in the blood [11]. Although elevated chemokine levels trigger trafficking of monocytes/macrophages, dendritic cells, natural killer cells, and lymphocytes to the CNS of infected patients, such molecules are essential for development of protective immunity against viral infections [30]. However, immune cells that have infiltrated the CNS may produce high levels of cytokines and chemokines upon stimulation with the EV71 virus. Such excessive chemokine production may be ultimately detrimental, exacerbating virus-induced inflammation and pathology by exaggerating the cell-mediated response. In contrast, no apparent correlation was evident in critical cases between cytokine levels and inflammatory markers. It may be caused by disorders triggered by the inflammatory response. Both a systemic inflammatory response and CNS inflammation may adversely affect prognosis, and both may play important roles in the development and progression of critical symptoms.

## Conclusions

Both inflammatory responses and disorders triggered by inflammation play important roles in the pathogenesis of severe EV71 infection. Systemic inflammation, indicated by elevated plasma IL-6 and IL-8 levels and higher WBC counts, may be a predictor of critical illness. Correlations between CSF cytokine levels and other markers of inflammation were confirmed in severely ill cases, but not in critically ill cases overwhelmed by inflammatory responses. More evidence, especially from case–control studies and animal models of EV71, is needed to determine if current interventions featuring IVIG and glucocorticoid improve the prognoses of severely ill patients. Our present study affords a new perspective on the pathogenesis of severe EV71 infection, and may facilitate design of novel interventions for patients with severe infections.

## Abbreviations

EV71: Enterovirus 71
IVIG: Intravenous immunoglobulin
CSF: Cerebrospinal fluid
CNS: Central nervous system
HFMD: Hand, foot, and mouth disease
NHFPC: National health and family planning commission
PE: Pulmonary edema
IL: Interleukin
WBC: White blood cell
CA16: Coxsackievirus A16
TNF-α: Tumor necrosis factor α
IP-10: Interferon gamma-induced protein 10
RANTES: Regulated on activation, normal T cell expressed and secreted chemokines
MCP-1: Monocyte chemotactic protein 1
MIG: Monokine induced by IFN-γ
SPSS: Statistical package for the social sciences
